# The Signal Peptidase FoSpc2 Is Required for Normal Growth, Conidiation, Virulence, Stress Response, and Regulation of Light Sensitivity in Fusarium odoratissimum

**DOI:** 10.1128/spectrum.04403-22

**Published:** 2023-06-27

**Authors:** Shuai Yang, Yanghong Zhuo, Yaqi Lin, Meimei Huang, Wei Tang, Wenhui Zheng, Guodong Lu, Zonghua Wang, Yingzi Yun

**Affiliations:** a State Key Laboratory of Ecological Pest Control for Fujian and Taiwan Crops, Ministerial and Provincial Joint Innovation Centre for Safety Production of Cross-Strait Crops, Fujian Agriculture and Forestry University, Fuzhou, China; b Institute of Oceanography, Minjiang University, Fuzhou, China; c Fujian Key Laboratory for Monitoring and Integrated Management of Crop Pests, Fuzhou, China; USDA-ARS-NPRL

**Keywords:** *Fusarium odoratissimum*, signal peptidase, FoSpc2, extracellular enzymes, light response

## Abstract

Signal peptidase (SPase) is responsible for cleavage of N-terminal signal peptides in most secretory precursor proteins and many membrane proteins during maturation. In this study, we identified four components of the SPase complex (FoSec11, FoSpc1, FoSpc2, and FoSpc3) in the banana wilt fungal pathogen Fusarium odoratissimum. We proved that interactions exist among the four SPase subunits by bimolecular fluorescence complementation (BiFC) and affinity purification and mass spectrometry (AP-MS) assays. Among the four SPase genes, *FoSPC2* was successfully deleted. *FoSPC2* deletion caused defects in vegetative growth, conidiation, and virulence. Loss of *FoSPC2* also affected the secretion of some pathogenicity-related extracellular enzymes, suggesting that SPase without FoSpc2 may have a lower efficiency in managing the maturation of the extracellular enzymes in *F. odoratissimum*. In addition, we found that the Δ*FoSPC2* mutant had increased sensitivity to light, and the colonies of the mutant grew faster under all-dark conditions than under all-light conditions. We further observed that deletion of *FoSPC2* affected expression of the blue light photoreceptor gene *FoWC2*, leading to cytoplasmic accumulation of FoWc2 under all-light conditions. Since FoWc2 has signal peptides, FoSpc2 may regulate the expression and subcellular localization of FoWc2 indirectly. Contrary to its response to light, the Δ*FoSPC2* mutant displayed a significant decreased sensitivity to osmotic stress, and culturing the mutant under osmotic stress conditions restored both the localization of FoWc2 and light sensitivity of the Δ*FoSPC2*, suggesting that a cross talk between osmotic stress and light response pathways in *F. odoratissimum* and FoSpc2 takes part in these processes.

**IMPORTANCE** In this study, we identified four components of SPase in the banana wilt pathogen Fusarium *odoratissimum* and characterized the SPase FoSpc2. Loss of *FoSPC2* affected the secretion of extracellular enzymes, suggesting that SPase without FoSpc2 may have a lower efficiency in managing the maturation of the extracellular enzymes in *F. odoratissimum*. In addition, this is the first time that we have found a relationship between the SPase and fungal light response. Deletion of *FoSPC2* resulted in decreased sensitivity to the osmotic stresses but with increased sensitivity to light. Continuous light inhibited the growth rate of the Δ*FoSPC2* mutant and affected the cellular localization of the blue light photoreceptor FoWc2 in this mutant, but culturing the mutant under osmotic stress both restored the localization of FoWc2 and eliminated the light sensitivity of the Δ*FoSPC2* mutant, suggesting that loss of *FoSPC2* may affect a cross talk between the osmotic stress and light response pathways in *F. odoratissimum*.

## INTRODUCTION

Protein secretion is a critical part of the physiology and virulence of fungal pathogens. Translocation across the membrane of the endoplasmic reticulum (ER) initiates protein transit through the secretory pathway (1), and this translocation process, which occurs either cotranslationally or posttranslationally, is triggered by signal peptides (SPs), short amino-terminal extension sequences that direct newly synthesized precursor proteins to translocation sites (or translocons) (2). Therefore, SPs work as “zip code” marking proteins destined to function in an extracytoplasmic location and directing them to a specific secretion pathway (3). Once the majority of the precursor proteins are translocated into the ER, the SPs are cleaved from the precursor proteins by signal peptidase complex, allowing for release from the membrane and correct folding of the mature protein (4).

Signal peptidase (SPase) is an evolutionarily conserved protease that was identified in all orders of life. In eukaryotes, SPase systems are located in the ER, mitochondria, and chloroplasts. In prokaryotes, SPases are classified into three groups: SPases I, II, and IV (3). Eukaryotic ER SPase is a heterooligomer consisting of membrane protein subunits, all of which are conserved from yeast to animals (5). Mammalian signal peptidase has been purified from dog pancreas microsomes as a complex of five different subunits, which are termed Spc12, Spc18, Spc21, Spc22/23, and Spc25 (4). In budding yeast (Saccharomyces cerevisiae), the SPase complex consists of four subunits: Spc1, Spc2, Spc3, and Sec11. Sec11 is homologous to canine Spc18 and Spc21; Spc1 and Spc2 are homologs of canine Spc12 and Spc25, respectively; and Spc3 is a homolog of canine SPC22/23 (6).

In S. cerevisiae, both Spc3 and Sec11 are single-pass membrane proteins, with the majority of the proteins being located in the ER lumen, and they are essential for yeast cell viability. Spc3 and Sec11 are required for the catalytic activity of the SPase complex, and deletion of either *SPC3* or *SEC11* leads to the loss of signal peptidase activity both *in vivo* and *in vitro* (6–8). Both Spc1 and Spc2 are nonessential for cell viability and signal peptide cleavage under normal growth conditions. A recent study demonstrated that Spc1 regulates the signal peptidase-mediated processing of membrane proteins by sharpening substrate sorting for SPase and protecting the transmembrane segments of membrane proteins from being cleaved by SPase (5). Spc2, but not Spc1, is important for signal peptidase activity and cell viability at high temperatures. At 42°C, *Spc2* mutants display an accumulation of precursors of secretory proteins *in vivo* and reduced cell viability (1, 9). Until now, studies on fungal SPase have been conducted only with S. cerevisiae, and little attention has been given to the biofunctions and mechanism of the SPase complex in other fungi, especially filamentous fungi.

The soilborne fungal pathogen Fusarium oxysporum f. sp. *cubense* (Foc) causes Fusarium wilt of banana, which seriously threatens banana production globally (10). Four races of Foc were identified based on their pathogenicity to reference host cultivars, and Foc tropical race 4 (FocTR4), among the four races, has the strongest virulence and can infect almost all commercial banana cultivars, including the Cavendish banana, which accounts for 28% of local consumption as well as 15% of export products (11). Several studies have found that Foc has a polyphyletic origin; therefore, Maryani et al. recently revised the taxonomy of Foc and designated Foc TR4 as Fusarium odoratissimum (12). Fusarium pathogens use both general and pathogen-specific mechanisms to invade their hosts. General virulence factors may include components of cellular signaling pathways, which are often required for proper development, and fungal enzymes used for degradation of plant cell walls; specific virulence factors may include host-specific toxins and secreted effectors (13). In *F. odoratissimum*, an effector family designated the Six (secreted in xylem) effectors, has been identified as special virulence factors in the pathogenicity of *F. odoratissimum* (14). Secretion of both general and pathogen-specific virulence-related factors is important, but we lack the knowledge of the elements that participate in the first stages of this process. In this study, we identified the various SPase complex subunits in *F. odoratissimum*, demonstrated the interaction relationships among the subunits, and then characterized *SPC2*, which encodes one subunit of the SPase complex in S. cerevisiae. We found that FoSpc2 mediates not only protein secretion but also environmental stress responses and participates in the regulation of vegetative growth, reproduction, and pathogenicity of *F. odoratissimum*.

## RESULTS

### Four genes encoding the four components of the signal peptidase complex were identified in *F. odoratissimum*.

**FIG 1:**
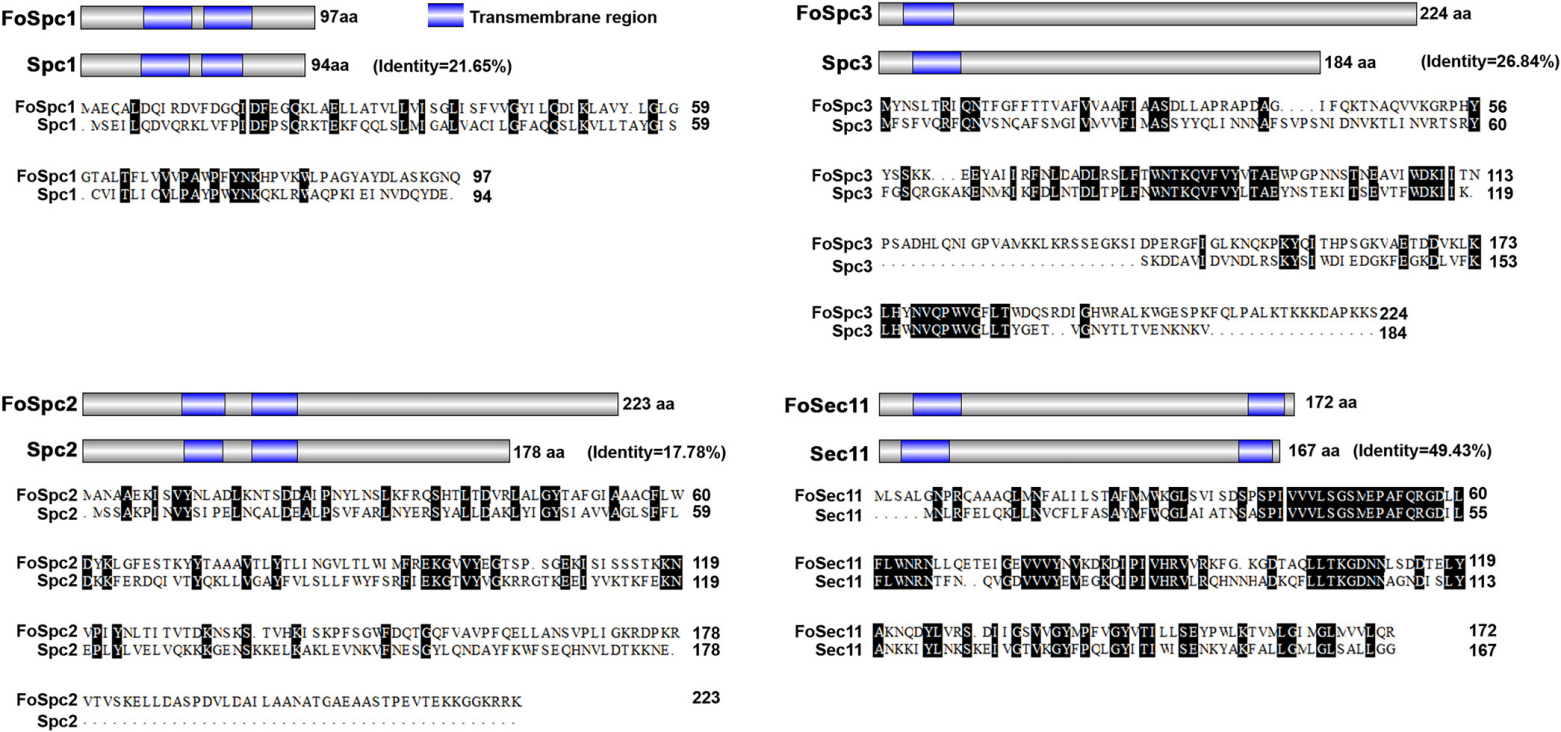
Domain analysis and multiple sequence alignment of signal peptidase subunits (Spc1, Spc2, Spc3, and Sec11) in S. cerevisiae and *F. odoratissimum*.

The signal peptidase (SPase) complex in S. cerevisiae contains four components, and the amino acid sequences of these four proteins were used to identify the corresponding homologs in *F. odoratissimum* by protein BLAST analysis (https://blast.ncbi.nlm.nih.gov/Blast.cgi) at the NCBI database. Four genes, FOIG_08844 (*FoSPC1*), FOIG_05064 (*FoSPC2*), FOIG_05783 (*FoSPC3*), and FOIG_03977 (*FoSEC11*), were found to encode the four components of the SPase complex in *F. odoratissimum*. In S. cerevisiae, Spc3 and Sec11 are required for the catalytic activity of SPase and are encoded by essential genes. The levels of protein identity between FoSec11 and Sec11, FoSpc3 and Spc3, FoSpc2 and Spc2, and FocSpc1 and Spc1 are 49.43%, 26.84%, 17.78%, and 21.65%, respectively. All the SPase proteins in S. cerevisiae and *F. odoratissimum* have transmembrane domains, and the number and distribution of the transmembrane domains in the proteins were similar between the corresponding protein homologs in these two fungi (Fig. 1).

### The interaction relationships among FoSpc3, FoSec11, FoSpc1, and FoSpc2 were identified by BiFC and AP-MS in *F. odoratissimum*.

**FIG 2:**
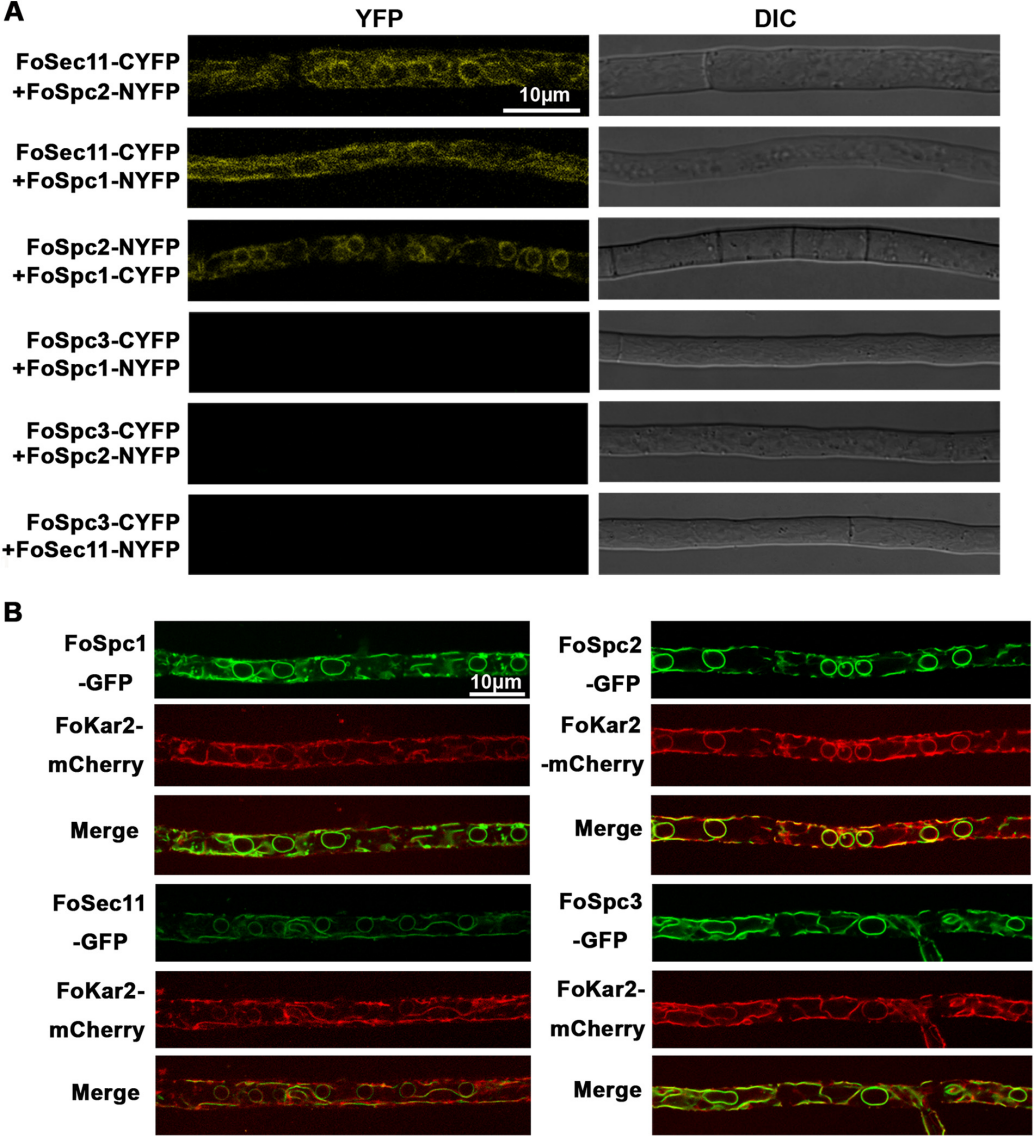
The cellular localization and interaction relationship of SPase components in *F. odoratissimum*. (A) BiFC assay for the interaction relationship of FoSec11, FoSpc2, FoSpc3, and FoSec11. YFP signals were observed in the strains expressing Sec11-CYFP and FoSpc2-NYFP, FoSec11-CYFP and FoSpc1-NYFP, and FoSpc2-NYFP and FoSpc1-CYFP vectors. YFP signals were not observed in those expressing FoSpc3-CYFP and FoSpc1-NYFP, FoSpc3-CYFP and FoSpc2-NYFP, or FoSpc3-CYFP and FoSec11-NYFP. Bar = 10 μm. (B) FoSpc1-GFP, FoSpc2-GFP, FoSec11-GFP, and FoSpc3-GFP can colocalize with the ER marker FoKar2-mCherry. Bar = 10 μm.

To demonstrate the nature of the SPase complex in *F. odoratissimum*, we identified the interaction relationship among FoSec11, FoSpc1, FoSpc2, and FoSpc3 by bimolecular fluorescence complementation (BiFC) assay. As shown in Fig. 2A, it is obvious that yellow fluorescent protein (YFP) signals concentrated at the cytoplasm of growing hyphal cells cotransformed with FoSec11-CYFP and FoSpc2-NYFP, FoSec11-CYFP and FoSpc1-NYFP, or FoSpc2-NYFP and FoSpc1-CYFP (C represent the C-terminal of YFP, and N represent the N-terminal of YFP), but we could not find any YFP signal in the hyphal cells cotransformed with FoSpc3-CYFP/NYFP and any of the other 3 components. Moreover, the YFP signals observed in the BiFC assays suggest that FoSec11, FoSpc1, and FoSpc2 may localize in the ER of the fungal cells (Fig. 2A). To verify these observations, we tagged the target genes (*FoSEC11*, *FoSPC1*, *FoSPC2*, and *FoSPC3*) with green fluorescent protein (GFP) and cotransformed each of them with the mCherry-tagged ER marker gene construct *FoKAR2-mCherry* into the wild-type strain. We observed that FoSec11-GFP, FoSpc1-GFP, FoSpc2_GFP, and FoSpc3-GFP clearly colocalized with FoKar2-mCherry (Fig. 2B), confirming that four SPase complex subunits are localized at the ER in *F. odoratissimum*.

Since we did not prove the interaction relationship among the FoSpc3 with the other three signal peptidase subunits (FoSpc1, FoSpc2, and FoSec11) by BiFC assays, we further used affinity purification and mass spectrometry (AP-MS) experiments to identify the relationships among the four signal peptidase subunits. FoSpc2 and FoSpc3 were fluorescently labeled with GFP, and the resulting transformants were used for protein extraction. In addition, the strain transformed only with GFP was used as a control. As shown below in Table 1, all signal peptidase subunits can be captured in FoSpc2 and FoSpc3 immunoprecipitation proteins, so we infer that the four signal peptidase subunits act as a complex, which is similar to their homologs in S. cerevisiae.

**TABLE 1:** Signal peptidase subunits in Fusarium odoratissimum identified by coimmunoprecipitation of FoSpc2 and FoSpc3

Protein identified by coimmunoprecipitation	Total spectral count/total protein coverage (%)^*a*^	
	FoSpc2-GFP	FoSpc3-GFP
FoSpc1	2/27	1/13
FoSpc2	16/52	12/51
FoSpc3	10/42	8/33
FoSec11	11/60	7/53

a Determined by GFP-trap + MS analysis.

### Deletion of *FoSPC2* affects the vegetative growth and conidiation of *F. odoratissimum*.

To study the biofunctions of the SPase complex in *F. odoratissimum*, we attempted to generate single-gene-deletion mutants for *FoSPC1*, *FoSPC2*, *FoSPC3*, and *FoSEC11* by a homologous recombination strategy. However, we could obtain gene deletion mutants only for *FoSPC2*, not for *FoSPC1*, *FoSPC3*, or *FoSPC11*, in *F. odoratissimum* after several trials. Three independent Δ*FoSPC2* mutants (Δ*FoSPC2-2*, Δ*FoSPC2-6*, and Δ*FoSPC2-9* mutants) were further subjected to Southern blotting (see Fig. S1 in the supplemental material) to confirm the gene deletions, and we used the Δ*FoSPC2-6* mutant as the progenitor strain for construction of the complemented Δ*FoSPC2-C* strain.

**FIG 3:**
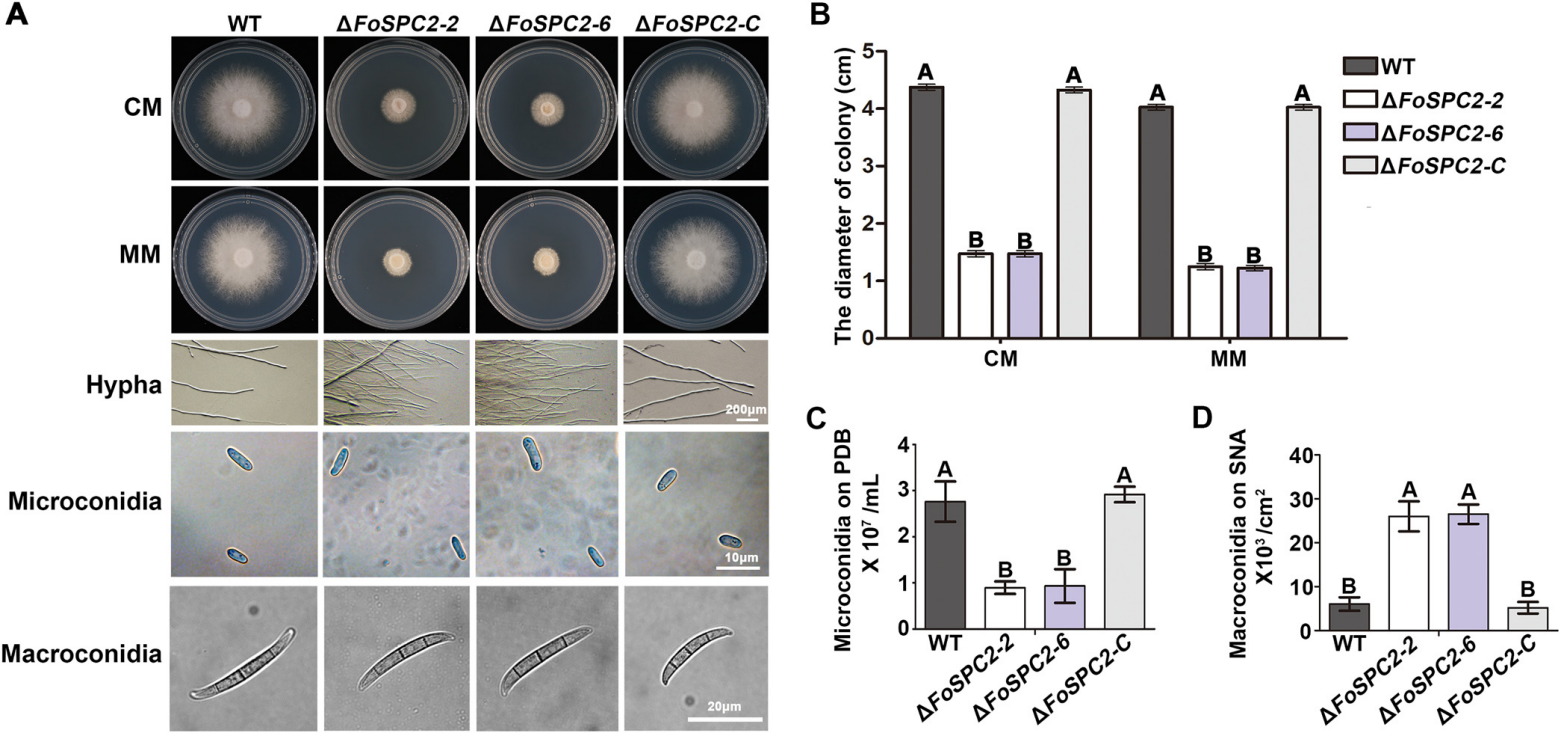
FoSpc2 is required for negative growth and conidiation of *F. odoratissimum*. (A) Colonies of the WT, two deletion mutants of *FoSPC2* (Δ*FoSPC2-2* and Δ*FoSPC2-6*), and the complementation strain Δ*FoSPC2-*C grown on CM for 3 days. The hyphae, microconidia, and macroconidia of these strains were observed under a microscope. (B) Statistical analysis of colony diameters of the indicated strains on CM and MM plates. (C and D) Statistical analysis of the production of microconidia (C) and macroconidia (D) from the above. Error bars represent the standard deviations (SD) for three replicates, and the same letter above the bars indicate nonsignificant differences at a *P* value of ≥0.01.

To evaluate the function of FoSpc2 in the regulation of vegetative growth in *F. odoratissimum*, the wild-type, Δ*FoSPC2* mutant, and complemented strains were grown on complete medium (CM) solid-agar plates for 3 days. The Δ*FoSPC2* mutant strains were observed to have smaller colonies than the wild-type and complemented strains (Fig. 3A and B). In addition, we found that the aerial hyphae of the Δ*FoSPC2* mutants were denser than those of the wild-type and complemented strains, but the three strains still had similar hyphal morphology (Fig. 3A).

Conidia, including microconidia and macroconidia, play important roles in the infection processes of *F. odoratissimum* (15). To assess the role of FoSpc2 in fungal conidiation, we harvested and analyzed the quantity of microconidia and macroconidia produced by the wild-type, Δ*FoSPC2*, and Δ*FoSPC2-C* strains cultured in potato dextrose broth (PDB) liquid medium and on Spezieller Nährstoffarmer agar (SNA) plates, respectively. We found that the quantity of microconidia produced by Δ*FoSPC2* was significantly low compared to those from the wild-type and complemented strains, while the quantity of macroconidia produced by the mutant was significantly higher than those from the wild-type and complemented strains (Fig. 3C and D). We further observed the morphology of the conidia from the various strains and found no obvious difference in the shapes and sizes of both kinds of conidia among the different strains (Fig. 3A). These results demonstrate that FoSpc2 is important for vegetative growth and conidium production in *F. odoratissimum* but does not influence conidial shapes and sizes during conidiation.

### Deletion of *FoSPC2* causes a defect in the pathogenicity of *F. odoratissimum*.

**FIG 4:**
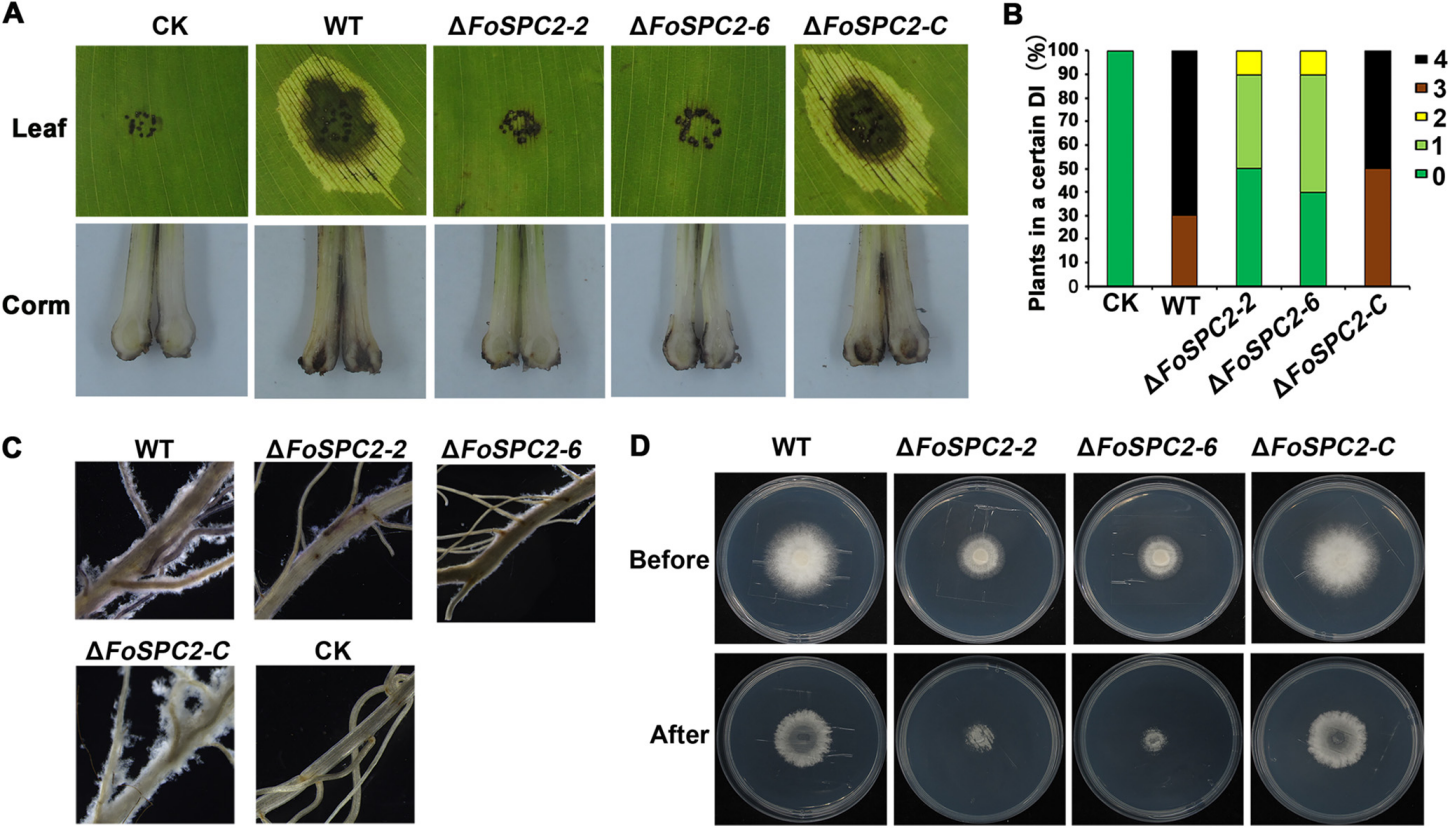
Deletion of *FoSPC2* affected the pathogenicity of *F. odoratissimum*. (A) Virulence analysis of the WT, two deletion mutants of *FoSPC2* (Δ*FoSPC2-2* and Δ*FoSPC2-6*), and the complementation strain (Δ*FoSPC2-C*) in banana leaves and corms. (B) The percentage of plants in each disease index exhibiting corm symptoms was scored: 0, no browning in the corm; 1, 1% to 20% browning area in the corm; 2, 20% to 40% browning area in the corm; 3, 40% to 60% browning area in the corm; 4, 60% to 100% browning area in the corm. (C) Penetration of the above-mentioned strains into the cellophane membrane. After removing colonies of the above-mentioned strains grown over cellophane membranes on MM (before) for 3 days, the plates were incubated for 2 days to examine hyphal growth (after). (D) Mycelium adhesion to banana roots seen after inoculating conidia from the above-mentioned strains on banana roots for 2 days.

To evaluate the role of FoSpc2 in the pathogenicity of *F. odoratissimum*, the wild-type, Δ*FoSPC2*, and Δ*FoSPC2-C* strains were inoculated on the roots or leaves of Cavendish bananas. At 2 months postinoculation, we observed vascular discoloration on the corms of the banana plantlets inoculated with the wild-type and complemented strains; however, the banana plants inoculated with the Δ*FoSPC2* mutant had only slight necrotic symptoms on the corms. Additionally, the banana leaves inoculated with the wild-type and Δ*FoSPC2-C* strains displayed obvious necrosis at the inoculation sites, while the leaves inoculated with the mutant strains showed no scab symptoms (Fig. 4A and B).

The infection processes of *F. odoratissimum* include root recognition, root surface adhesion and colonization, penetration of the root cortex, and hyphal proliferation within the xylem vessels (16). To explore the reasons for the decreased virulence of the Δ*FoSPC2* strain, we evaluated the ability of the Δ*FoSPC2* strain to adhere to the root surface by immersing the banana roots in conidial suspensions of the wild-type, Δ*FoSPC2*, and Δ*FoSPC2-C* strains. As shown in Fig. 4C, the wild-type and Δ*FoSPC2-C* strains efficiently adhered to the roots and formed a dense hyphal network covering most of the root surface at 48 h postinoculation, while two Δ*FoSPC2* mutant strains showed significantly reduced ability to adhere to and grow on the root surfaces, suggesting that deletion of *FoSPC2* affected the adhesion ability of *F. odoratissimum*, which may be the reason for the decreased virulence of the Δ*FoSPC2* mutant. Cellophane membrane is widely used to investigate the development of infection structures in plant-pathogenic fungi (17). The ability of the Δ*FoSPC2* strain to penetrate cellophane membrane was investigated, and we discovered that the ability of the mutant strain to penetrate the cellophane membrane was similar to the penetration ability of the wild-type and Δ*FoSPC2-C* strains (Fig. 4D), indicating that deletion of *FoSPC2* does not influence the penetration ability of *F. odoratissimum*.

### Deletion of *FoSPC2* affects the secretion of extracellular enzymes by *F. odoratissimum*.

**FIG 5:**
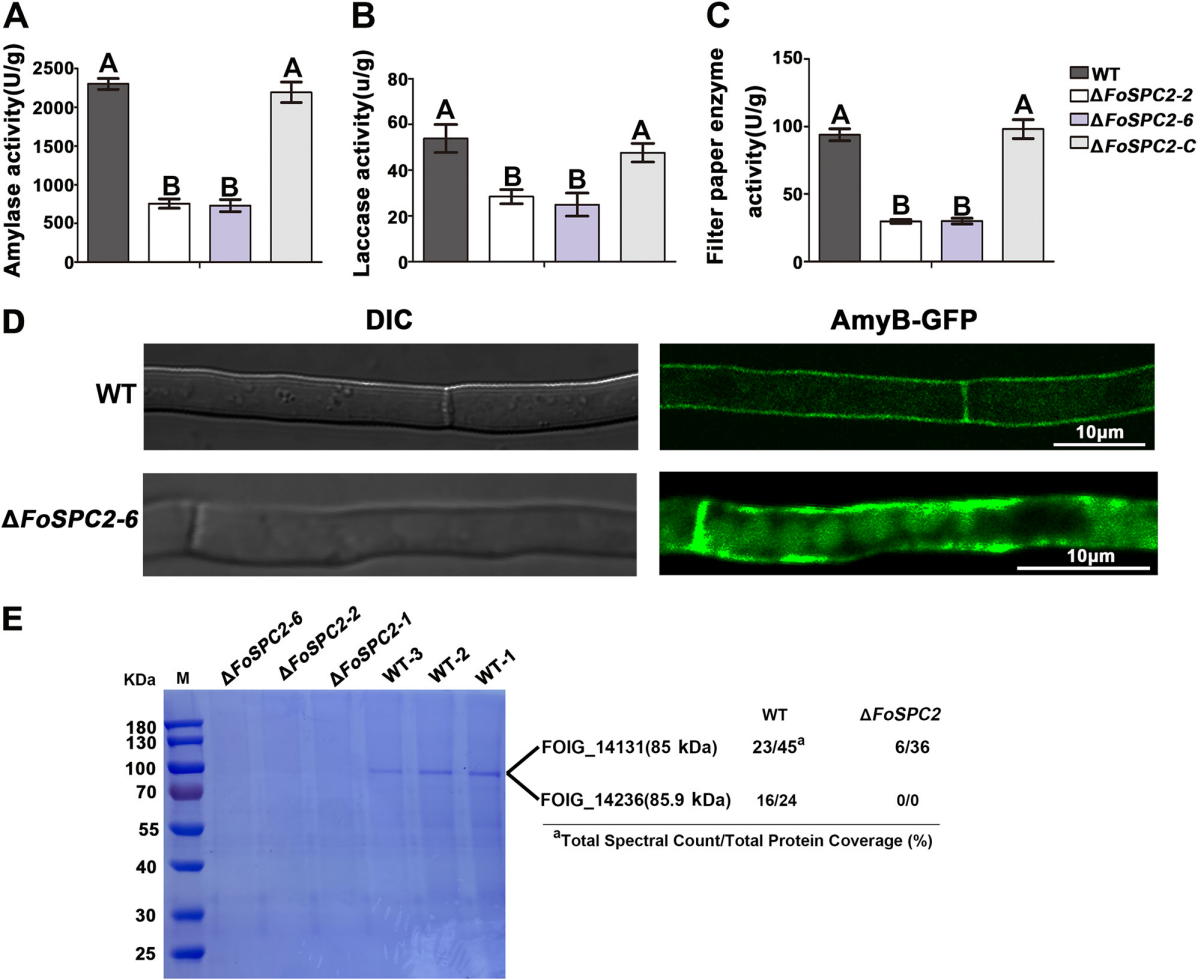
FoSpc2 is required for the secretion of extracellular enzymes. The activities of amylase (A), laccase (B), and filter paper enzyme (C) were detected using the DNS method in the WT, two deletion mutants of *FoSPC2* (Δ*FoSPC2-2* and Δ*FoSPC2-6*), and the complementation strain (Δ*FoSPC2-C*). Error bars represent the SD for three replicates, and the same letters above the bars indicate nonsignificant differences at a *P* value of ≥0.01. (D) Subcellular localization of AmyB-GFP in the WT and Δ*FoSPC2* deletion mutant strains. Bar = 10 μm. (E) The secreted proteins from the WT and Δ*FoSPC2* strains were detected by SDS-PAGE, and proteins in the different bands were identified by MS.

Secreted cell wall-degrading enzymes (CWDEs), e.g., cellulase, xylanase, pectinase, and amylase, are important for overcoming plant cell walls in the infection process of *F. odoratissimum* (18). To explore whether loss of FoSpc2 affects the secretion of CWDEs, we evaluated the activities of laccase, amylase, and filter paper enzyme from the wild-type, Δ*FoSPC2*, and Δ*FoSPC2-C* strains. Previous studies have demonstrated that filter paper can be used as a substrate to detect total cellulase activity, including endoglucanase, exoglucanase, and β-glucosidase, so filter paper activity (FPA) can represent the activity of total cellulase (18). Our results showed that the activities of these extracellular hydrolases are significantly decreased in the Δ*FoSPC2* strains compared to the wild-type and complemented strains (Fig. 5A to C), suggesting that deletion of *FoSPC2* affects the secretion of CWDEs in *F. odoratissimum*. To further confirm this, we observed the cellular localization of alpha-amylase (AmyB; FOIG_14103) in the wild-type and Δ*FoSPC2* strains and found that the AmyB-GFP signals localized on the cell membrane and septa of the wild-type strain, while in the Δ*FoSPC2* mutant, AmyB-GFP signals accumulated not only as spots on the cell membrane but also dispersed in the cytoplasm (Fig. 5D). The precursor of AmyB contains the signal peptide, indicating that deletion of *FoSPC2* may inhibit the secretion of AmyB by affecting its maturation.

To further analyze the role of FoSpc2 in the secretion of extracellular enzymes, we collected the secreted proteins of the wild-type and Δ*FoSPC2* strains from the supernatant of the fungal liquid culture; the amount of supernatant used for protein extraction was adjusted based on the mycelial dry mass obtained from the cultures. The secreted proteins were detected by SDS-PAGE and staining with Coomassie brilliant blue; we found a bright protein band between 70 and 100 kDa in the wild-type samples, which did not appear in the Δ*FoSPC2* samples (Fig. 5E), and then we cut this bright band and the corresponding place on the same gel from the Δ*FoSPC2* samples. Through protein identification by mass spectrometry, we found that two 85-kDa proteins encoded by FOIG_14131 and FOIG_16236 accumulated much more in the wild-type samples and appeared in a much smaller amount in the Δ*FoSPC2* samples (Fig. 5E). FOIG_14131 and FOIG_16236 are predicted to encode catalase-peroxidases, which contain signal peptides in the N terminus, suggesting that FoSpc2 takes part in the maturation process of FOIG_14131 and FOIG_16236 proteins. The above results suggested that FoSpc2 is required for the secretion of extracellular enzymes, and SPase without FoSpc2 may have a lower efficiency in managing the maturation of some extracellular enzymes in *F. odoratissimum*.

In S. cerevisiae, SPase is responsible for the cleavage of signal peptides in most secretory precursor proteins and many membrane proteins during maturation. Kar2 acted as an ER molecular chaperone, and the signal peptide of pre-Kar2 (the precursor of Kar2) is cleaved by the SPase (8). In *F. odoratissimum*, we transformed the coding sequence of FoKar2 linked with the red fluorescent protein (RFP) sequence in the C terminus into the wild-type and Δ*FoSPC2* strains and found that the cellular localization of FoKar2-RFP in the Δ*FoSPC2* did not show a big difference from that in the wild type (Fig. S2A). We further detected the protein bands of pre-FoKar2 and FoKar2 in the wild-type and Δ*FoSPC2* strains by Western blotting and found only one same-sized band of FoKar2-RFP in the wild-type and Δ*FoSPC2* strains (Fig. S2B), suggesting that SPase without FoSpc2 is still functional to cut the signal peptide of pre-FoKar2 in *F. odoratissimum.* In S. cerevisiae, Spc3 and Sec11 are essential for the activity of SPase, while Spc2 is nonessential for cell growth and enzyme activity but is important for enzyme activity and cell viability at elevated temperatures. In *F. odoratissimum*, we found that deletion of *FoSPC2* did not affect the maturation of FoKar2 but affected the maturation of some extracellular enzymes; therefore, we inferred that FoSpc2 may be required for the activity of the SPase in some conditions.

### High osmotic pressure restores the growth defects of the Δ*FoSPC2* strain.

**FIG 6:**
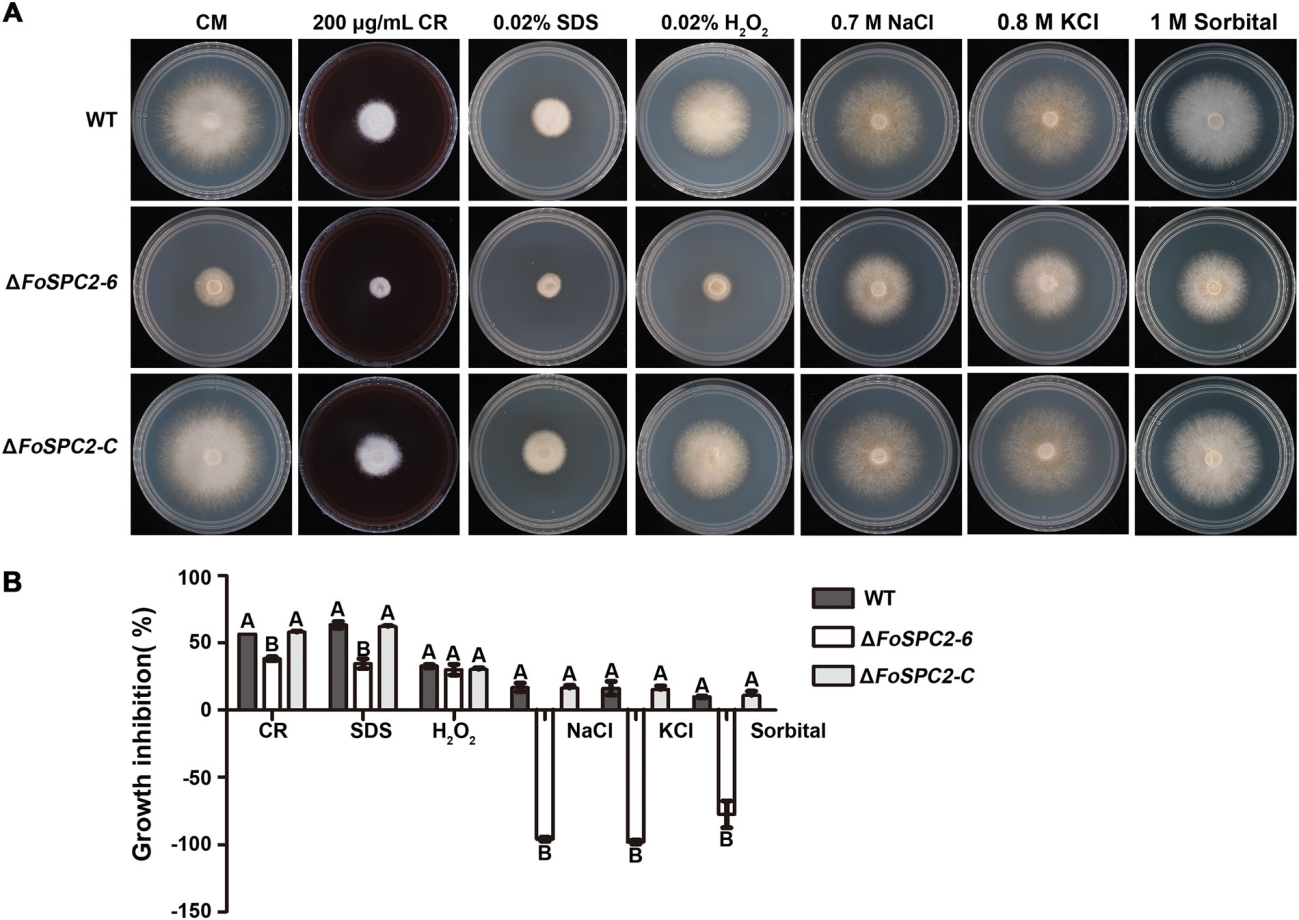
Sensitivity of the WT strain, the *FoSPC2* deletion mutant (Δ*FoSPC2-2*), and the complemented strain (Δ*FoSPC2-C*) under different stress conditions. (A) Strains were grown on CM without or with various stress inducers as indicated at 28°C for 3 days. (B) Statistical analysis of the colony diameters in panel A. The growth inhibition rate is relative to the growth rate of each untreated control. Error bars represent the SD for three replicates, and the same letters above the bars indicate nonsignificant differences at a *P* value of ≥0.01.

Protein secretion is essential for fungal apical growth, which provides the necessary materials and enzymatic platforms needed for tip cell wall expansion and growth (19). To determine whether FoSpc2 is involved in *F. odoratissimum* response to stresses, we investigated the growth of all the strains on medium containing stress-mimicking agents, including NaCl, H_2_O_2_, sodium dodecyl sulfate (SDS), and Congo red (CR). We found that the Δ*FoSPC2* mutant showed increased tolerance to the cell wall stress-inducing agent CR and the cell membrane stress-inducing agent SDS compared to the wild-type strain, but no difference was observed in stress tolerance to H_2_O_2_, an oxidative stress-inducing agent. However, the Δ*FoSPC2* mutant was observed to grow much better on CM plates containing a 0.7 M concentration of the osmotic stress agent NaCl, so we further inoculated each strain on CM plates containing KCl (0.8 M) and sorbitol (1 M) and similarly found that the Δ*FoSPC2* mutant grew better on the plates supplemented with KCl and sorbitol (Fig. 6A and B), confirming that high osmotic pressure partially restored the growth defects of the Δ*FoSPC2* strain.

### Deletion of *FoSPC2* leads to increased light sensitivity of *F. odoratissimum*.

**FIG 7:**
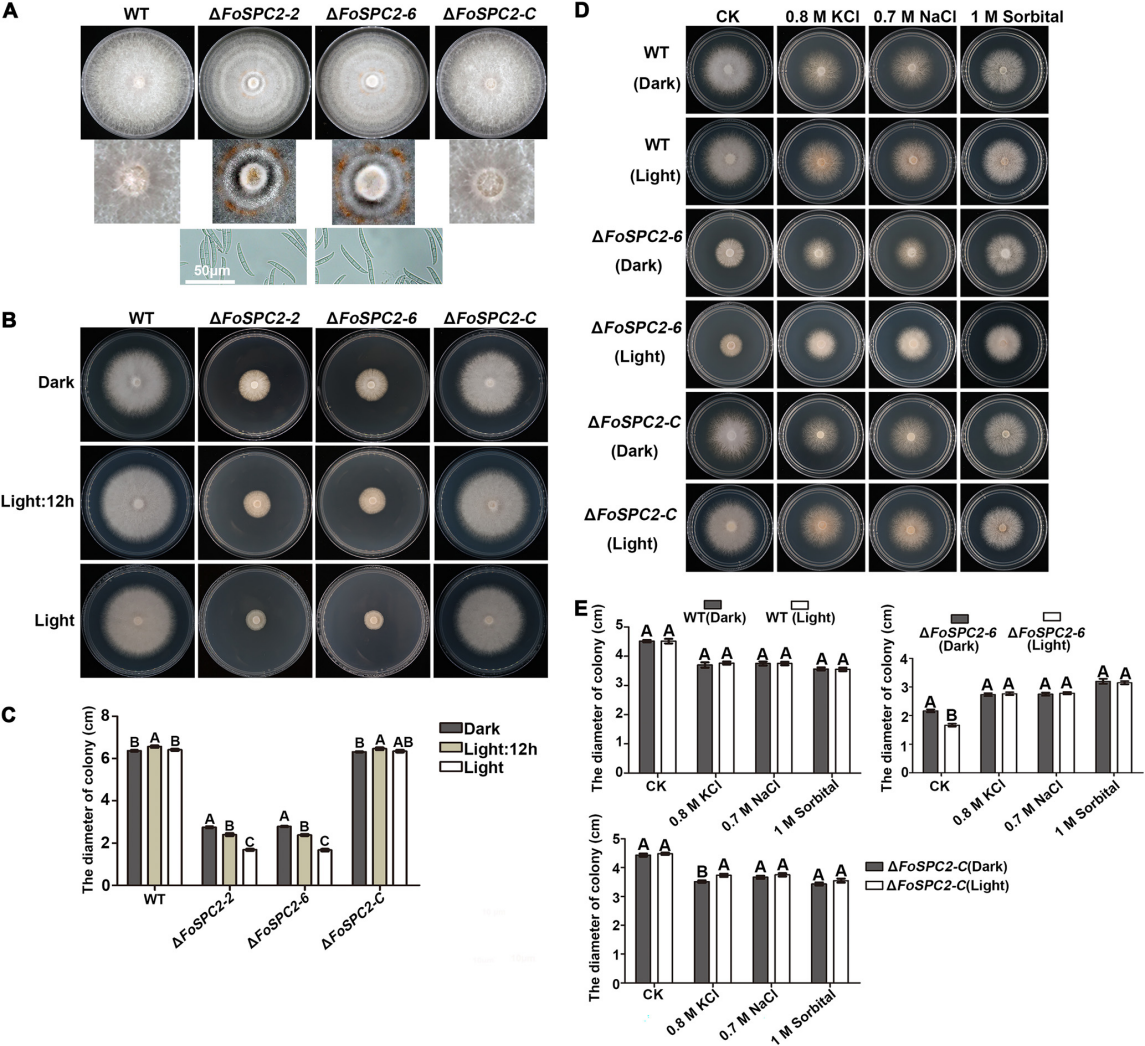
The growth rate of Δ*FoSPC2* was affected by light. (A) Colony morphology of the WT strain, the *FoSPC2* deletion mutant (Δ*FoSPC2-2* and Δ*FoSPC2-6*), and the complemented strain (Δ*FoSPC2-C*) cultured for 10 days in CM plates. (B) Colonies of the above-mentioned strains grown on CM for 5 days under 3 different light conditions (all dark, 12 h light, and all light). (C) Statistical analysis of colony diameters in panel B. (D) Colonies of the above-mentioned strains in CM with added KCl (0.8 M), NaCl (0.7 M), and sorbitol (1 M) under all-light and all-dark conditions. (E) Statistical analysis of colony diameters in panel D. Error bars represent the SD for three replicates, and the same letters above the bars indicate nonsignificant differences at a *P* value of ≥0.01.

When all the strains in this study were cultured for a long time (10 days) under a 12-h/12-h light/dark cycle, we noticed that the colonies of the Δ*FoSPC2* mutant showed a zonation phenomenon and accumulated orange spots containing numerous macroconidia at the central regions of the colonies, and these phenotypes were not seen in colonies of the wild-type and complemented strains (Fig. 7A), suggesting that the Δ*FoSPC2* mutant displays sensitivity to light different from that of the wild-type strain. To test the effect of light/dark stimulus on the growth of the Δ*FoSPC2* mutant, we cultured the various strains under light, dark, and 12-h/12-h light/dark conditions and observed that the Δ*FoSPC2* mutant grew fastest in the dark and faster in the 12-h/12-h light/dark cycle than in the light, while the growth rates of the wild-type and complemented strains were not altered under these three conditions (Fig. 7B and C). These results suggest that light inhibits the growth of Δ*FoSPC2* mutant.

Since our earlier findings showed that the Δ*FoSPC2* mutant also grows faster under high osmotic pressure, we became curious as to what the growth rate of the mutant would be when it was cultured in light or dark under high osmotic pressure. We therefore inoculated the wild-type, Δ*FoSPC2*, and complemented strains on CM plates supplemented with 0.8 M KCl, 0.7 M NaCl, and 1 M sorbitol, respectively. Interestingly, we found that the Δ*FoSPC2* mutant shows the same growth pattern under light/dark conditions in the presence of the osmotic stress agents (Fig. 7D and E), suggesting that osmotic stress restores the light sensitivity of the Δ*FoSPC2* strain.

### Deletion of *FoSPC2* affects the cellular localization of FoWc2 in *F. odoratissimum*.

**FIG 8:**
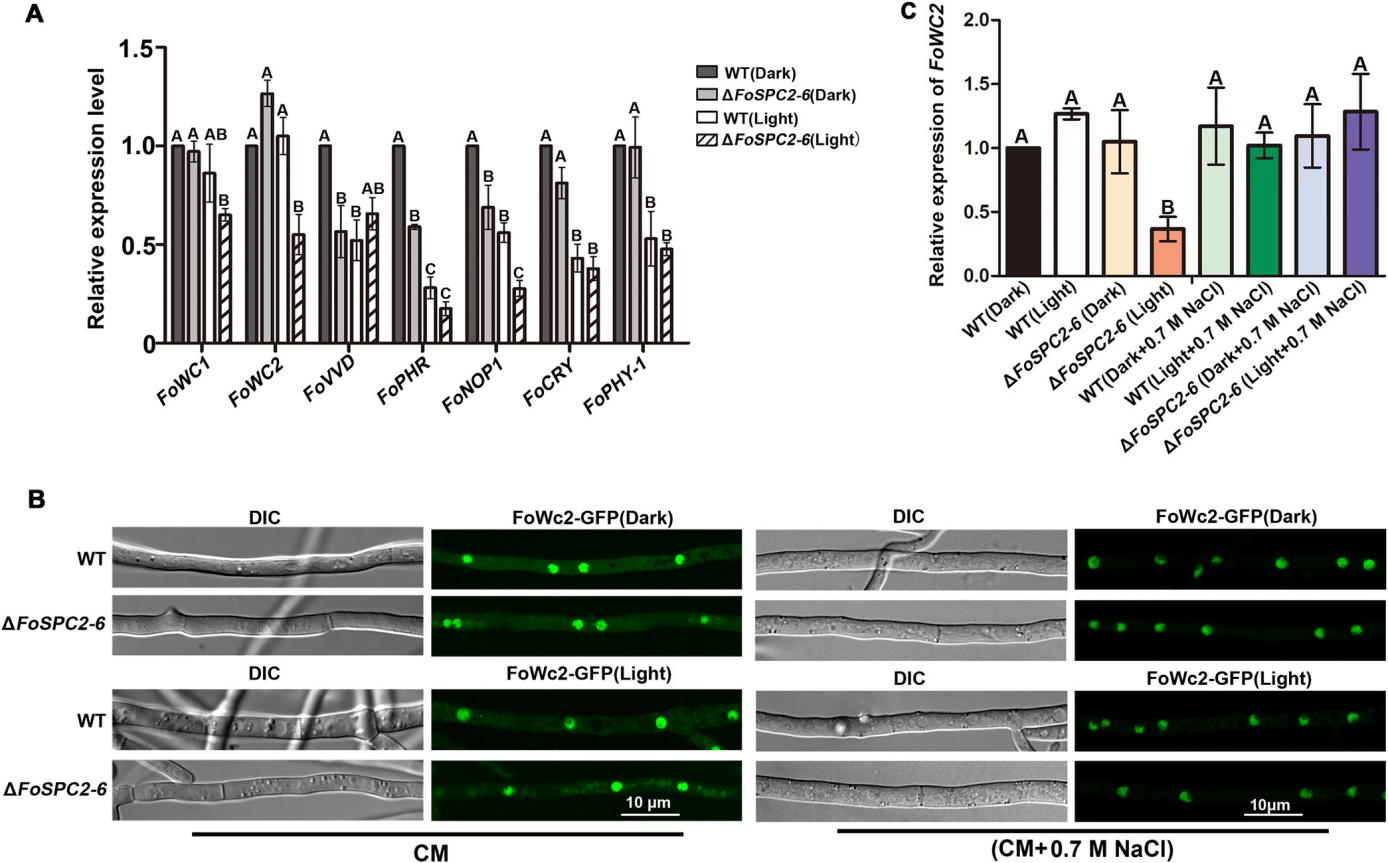
Deletion of *FoSPC2* affected the gene expression level and cellular localization of FoWc2 under different light conditions. (A) Comparison of photoreceptor gene expression in the WT strain with that in the Δ*FoSPC2* mutant under all-dark and all-light conditions. Error bars represent the SD for three replicates, and the same letters above the bars indicate nonsignificant differences at a *P* value of ≥0.01. (B) Subcellular localization of FoWc2-GFP in WT and the Δ*FoSPC2* strain under all-dark and all-light conditions in CM or CM supplemented with 0.7 M NaCl. Bar = 10 μm. (C) Relative expression of *FoWC2* in the wild-type and Δ*FoSPC2* strains under high osmotic stress between all-light and all-dark conditions. The same letters above the bars indicate nonsignificant differences at a *P* value of ≥0.01.

Fungi can sense different light levels using a series of photoreceptors (20). To demonstrate whether deletion of *FoSPC2* affects the functions of some photoreceptors, we first detected the gene expression levels of 7 photoreceptors in Δ*FoSPC2*, which include the blue-light photoreceptors *FoWC1* (FOIG_14437), *FoWC2* (FOIG_13125), *FoVVD* (FOIG_02260), *FoCRY1* (FOIG_12065) and *FoPHR* (FOIG_13229), the green-light photoreceptor *FoNOP1* (FOIG_02552), and the red-light photoreceptor *FoPHY1* (FOIG_12973). The results showed that the expression of *FoWC2* was significantly changed between light and dark conditions (Fig. 8A). Therefore, we further analyzed the cellular localization of FoWc2 in the wild-type and Δ*FoSPC2* strains. We observed that, under dark conditions, FoWc2-GFP mainly localized in the nucleus in both the wild-type strain and Δ*FoSPC2* mutant (Fig. 8B). Under light conditions, FoWc2-GFP still localized in the nucleus of the wild-type strain, but we noticed the accumulation of FoWc2-GFP as spots in the cytoplasm of the Δ*FoSPC2* mutant, although the GFP signal could still be observed in the nucleus of the mutant (Fig. 8B). The above results indicate that deletion of *FoSPC2* affects the cellular localization of FoWc2 in *F. odoratissimum.* We further observed FoWc2-GFP localization in the wild-type and Δ*FoSPC2* strains under high osmotic pressure and found that FoWc2-GFP localized only in the nucleus in both the wild-type andΔ*FoSPC2* strains under both dark and light conditions (Fig. 8B), supporting the idea that high-osmotic-stress conditions restore the localization defect of FoWc2 in the Δ*FoSPC2* strain under light conditions. Meanwhile, to test whether the expression of *FoWC2* was restored under high osmotic stress (0.7 M NaCl) between all-light and all-dark conditions, we extracted the total RNA from the corresponding mycelial tissues. The results of quantitative real-time PCR (qRT-PCR) showed that the expression of *FoWC2* was decreased in the Δ*FoSPC2* mutant under all-light conditions but was not significantly different between all-light and all-dark conditions under high osmotic stress (Fig. 8C). These results are consistent with the previous results that osmotic stress restores the light sensitivity of the Δ*FoSPC2* mutant.

## DISCUSSION

The N-terminal signal peptide extension is recognized as a feature common to most, but not all, secretory precursor proteins and many membrane proteins. Signal peptides are cleaved during translocation of the protein across the bilayer by a highly selective, membrane-bound peptidase (21). This signal peptidase (SPase) is found in prokaryotic and eukaryotic cells, but the biofunctions and mechanisms of action of SPase in fungal species other than budding yeast are unknown. In this study, we identified four genes encoding four different subunits of the SPase complex in the filamentous fungus *F. odoratissimum* and demonstrated the interaction relationship among them. Of the four SPase complex genes, we deleted only *FoSPC2* successfully, and the deletion of *FoSPC2* caused defects in the vegetative growth, reproduction, virulence, and stress responses of *F. odoratissimum* and also affected protein secretion, especially CWDE secretion.

In S. cerevisiae, *SPC3* and *SEC11* are essential genes, while *SPC1* and *SPC2* can be individually or collectively deleted (22). In *F. odoratissimum*, we could not obtain deletion mutants for *FoSPC3* or *FoSEC11* after screening many transformants in five independently repeated experiments; therefore, we inferred that *FoSPC3* and *FoSEC11* are also essential genes in *F. odoratissimum*. In S. cerevisiae, Spc1 is dispensable for growth, while in *Drosophila*, the homolog of *SPC1*, *SPASE12*, is an essential gene; its null alleles are lethal, and Spase12 was shown to function in mediating cell differentiation and development (23). In human cell lines, Spcs1, the homolog of Spc1, serves as a key host factor in the processing of viral proteins by interacting with the transmembrane (TM) domains of Japanese encephalitis virus as well as hepatitis C virus proteins (24, 25). Since we failed to delete *FoSPC1* in *F. odoratissimum* during this study, and homologs of *FoSPC1* have not been studied in other filamentous fungi, whether *FoSPC1* is also an essential gene could not be determined here and needs further confirmation. In S. cerevisiae, Spc2 is required for maximal signal peptidase activity under certain growth conditions, while in filamentous fungi, little is known about its homologs’ function (26). In *F. odoratissimum*, FoSpc2 was required for protein secretion and played important roles in vegetative growth, conidiation, virulence, and the external stress responses, suggesting that FoSpc2 displays species-specific biofunctions compared with Spc2 in S. cerevisiae.

Similar to other root-infecting fungi, *F. odoratissimum* persists in the soil for extended time periods, and then compounds exuded by the host plant trigger spore germination, followed by directed hyphal growth and penetration of the root (27). During this initial stage of infection, mycelial adherence to the surface of banana roots is important for penetration (28). In this study, we found that deletion of *FoSPC2* significantly affected mycelial adherence to the roots, which may be one of the reasons for the decreased virulence of the Δ*FoSPC2* mutant. In F. oxysporum f. sp. *lycopersici* (Fol), the orthologous MAPK (mitogen-activated protein kinase) protein Fmk1 is essential for tomato infection. Δ*FMK1* mutants were unable to adhere to and colonize tomato roots (28), and a significant reduction in pectinolytic activity was also found in the mutant. We also found that loss of *FoSPC2* results in decreased some CWDE activities in *F. odoratissimum*, so we inferred that secretion of extracellular CWDEs contributes to the adherence process of *F. odoratissimum* on the host roots.

While living in the soil or during colonization in the xylem, *F. odoratissimum* should adapt to the changed environmental stress from external conditions. In this study, we found that the Δ*FoSPC2* mutant showed decreased sensitivity to cell wall, cell membrane, and osmotic stresses but displayed sensitivity to oxidative stress similar to that of the wild-type strain. In filamentous fungi, apical secretion is essential for fungal apical growth at hyphal tips, which provides the necessary materials and enzymatic platforms needed for tip cell wall expansion and growth (19). We found here that deletion of *FoSPC2* affected protein secretion, so we hypothesized that deletion of *FoSPC2* may affect the cell wall and cell membrane architecture in *F. odoratissimum*, which leads to different responses of the Δ*FoSPC2* mutant to the environmental stresses.

Light is a very important signal that fungi perceive during interaction with the environment, and it regulates the fungal development and behavior and activates metabolic pathways (29). Fungi use blue light as the primary signal for photoreception and the white collar complex (WCC) as the primary photoreceptor system for the blue light. The WCC consists of the proteins white collar 1 (Wc-1) and white collar 2 (Wc-2) (20, 29). The molecular mechanisms of fungal photoreception have been explored in detail in Neurospora crassa (29). In N. crassa, Wc-1 and Wc-2 interact through their PAS (Per-Arnt-Sim) domains to form a WCC that binds the promoter of light-inducible genes; the WCC proteins are preferentially located in the nucleus, although Wc-2 is also observed in the cytoplasm and is more abundant than Wc-1, and nuclear localization of either Wc-1 or Wc-2 is not affected by light. The *WC-1* gene is induced by light, but *WC-2* is equally expressed in dark- and light-grown N. crassa mycelia (30, 31). However, in this study, we found that the expression level of *FoWC2* (the homolog of *WC-2* in *F. odoratissimum*) in the Δ*FoSPC2* mutant showed upregulation under all-dark conditions but downregulation under all-light conditions, and the cytoplasmic localization of FoWc2 was also affected by light in the Δ*FoSPC2* mutant (Fig. 8). Since FoWc-2 does not have a signal peptide, it is not the substrate of SPase, and therefore, we hypothesize that deletion of *FoSPC2* may influence the expression and cellular localization of FoWc2 indirectly in *F. odoratissimum*.

Although the subcellular localizations and functional mechanisms of Wc2 homologs and the WCC have not been studied in Fusarium species, the biofunctions of Wc-2 homologs have been investigated in Fusarium asiaticum and F. graminearum (32, 33). FoWc2 shares 99% protein similarity with FaWc2 and FgWc2; deletion of either *FaWC2* or *FgWC2* affected the fungal carotenoid biosynthesis and sexual reproduction but did not affect the growth and pathogenicity of the progenitor (32, 33). In our work, different light treatments affected the growth rate of the Δ*FoSPC2* mutant, suggesting that the Δ*FoSPC2* mutant had higher sensitivity to light than the *FaWC2* or *FgWC2* mutants. Therefore, we hypothesized that deletion of *FoSPC2* not only influenced FoWc2 but also affected some other components of the light response pathways in *F. odoratissimum*. In Aspergillus nidulans, the MAP kinase HogA was identified as a possible signaling factor downstream of red-light sensing (34). HogA is the key kinase in the high osmolarity glycerol (HOG) pathway, which regulates the hyperosmotic stress response of filamentous fungi. In the present study, interestingly, we found that osmotic stress can restore the light sensitivity of the Δ*FoSPC2* mutant, indicating that cross talk may also exist between the osmotic stress response and light response pathways in *F. odoratissimum* and that FoSpc2 takes part in these processes. To our knowledge, this is the first study to unveil the link between the SPase complex and fungal light response regulation, although the specific regulatory mechanism needs further exploration.

## MATERIALS AND METHODS

### Fungal strains and culture conditions.

Fusarium odoratissimum strain 58 was used as the wild-type (WT) strain for constructing various gene deletion mutants (35, 36). All strains were cultured on complete medium (CM) or minimal medium (MM) at 28°C for 3 days to measure the growth rate or assess colony morphology. For conidiation assays, microconidia were harvested from 2-day-old liquid potato dextrose broth (PDB) cultures, while macroconidia were cultured on 7-day-old solid Spezieller Nährstoffarmer agar (SNA) medium. These experiments were repeated three times independently.

### Construction of gene deletion and complementation mutants.

All SPase complex gene replacement constructs were generated by a double-joint PCR approach and transformed into protoplasts of WT strains (37). The primers used to amplify the upstream and downstream fragments of all the signal peptidases (including FoSpc1, 2, 3 and Sec11) are listed in Table S1. Transformants were first screened by PCR, and positive ones were further confirmed by Southern blotting. For gene complementation assays, all target genes (without stop codons), including their native promoter regions, were amplified by PCR, cloned into pKNT vectors using a one-step cloning kit (Vazyme Biotech, China), and transformed into the *FoSPC2* mutant (38).

### Pathogenicity assays.

For banana root infection, conidia were harvested from 2-day-old PDB culture and their concentrations were adjusted to 10^6^ conidia/mL in sterile distilled water. At the five-leaf stage, banana plantlets (Cavendish banana, AAA cultivar) were infected with conidia from the various fungal strains. Prior to the infection, the plant roots were wounded to promote the infection process. At 30 days postinfection, disease symptoms were observed as described by Li et al. (39). An internal scoring system was used to measure the area of discolored patches of individual plants (40). Leaf inoculation was performed using conidial suspensions (10^6^ spores/mL) as previously described (41). Disease progression was assessed at 5 days postinoculation (dpi). The experiments were repeated three times independently.

### Penetration and attachment assays.

For cellophane penetration experiments, conidia were harvested from 2-day-old PDB cultures and adjusted to 10^6^ spores/mL in sterile distilled water. Next, 10 μL conidial suspension was transferred to MM plates containing a layer of cellophane. After 3 days, the cellophane was removed with tweezers, and the plates were further incubated for 2 days to examine subsequent hyphal growth. For adhesion assays, the roots of the banana plantlets were placed in Erlenmeyer flasks containing 100 mL of conidial suspensions (10^6^ conidia/mL) and incubated for 2 days at 28°C and 120 rpm. Adhesion of the mycelia to the root surfaces of the plantlets was then observed (28). Both the cellophane penetration and root adhesion experiments were performed three times.

### Activity detection for extracellular hydrolases.

For extracellular hydrolase activity experiment, fresh mycelial blocks were inoculated with 100 mL of Czapek-Dox medium containing 2% bran for 7 days. The mycelia were then removed completely by filtration, and the culture filtrate was used to measure extracellular enzyme activity. The activities of amylase and the filter paper enzyme were determined using the 3,5-dinitrosalicylic acid (DNS) method with minor modifications as previously described (42, 43). Brief descriptions of the method follow.

For detection of the amylase activity, 1 mL 1% starch solution (0.1 M citric acid buffer, pH 5.6) was incubated at 40°C for 5 min, and then 0.5 mL filtrate was added. After incubation at 40°C for 30 min, 1 mL DNS reagent was added into the mixture to stop the reaction, and then the volume of the mixture was brought to 10 mL with sterilized water. The absorbance of the mixture was measured at 540 nm, and the amount of maltose in the mixture was calculated based on the standard curve of maltose absorbance at 540 nm. One unit of enzymatic activity is defined as 1 μg/min reducing maltose from the substrate at pH 5.6 and 40°C.

For detection of the filter paper enzyme activity, 50 mg filter paper was combined with 1 mL 0.1 M citric acid buffer solution (pH 5.0) and incubated at 50°C for 5 min. One milliliter of filtrate was added to the mixture and incubated at 50°C for 60 min, and then 1 mL DNS reagent was added to stop the reaction. Then, the volume of the mixture was brought to 10 mL with sterilized water, and the absorbance of the mixture was measured at 540 nm. The amount of glucose in the mixture was calculated based on the standard curve of glucose absorbance at 540 nm. One unit of enzymatic activity is defined as 1 μg/min reducing glucose from the substrate at pH 5 and 50°C. Inactivated enzyme solution was used as a blank control.

The activity of laccase was determined by using the 2,2′-azino-bis (3-ethylbenzothiazoline-6-sulfonic acid) (ABTS) method as previously described (44). Briefly, 2.7 mL acetic acid (HAc)-sodium acetate (NaAc) solution (pH 4.6) and 0.2 mL ABTS solution (1 mmol/L) were mixed and incubated for at 30°C 5 min, and the absorbance of the mixture was measured at 420 nm. Then, 200 μL filtrate was added, and after incubation for 3 min, the absorbance of the mixture was measured again at 420 nm. The activity can be calculated by the following formula: activity (units per liter) = (10^6^/ε_420_) × (vol_mixture_/vol_filtrate_) × (ΔOD/3); ε_420_ = 36,000 M^−1 ^cm^−1^. The dry weights of the harvested mycelia were measured to normalize the enzyme activities.

### AP-MS.

To identify signal peptide complex interactions in *F. odoratissimum*, the wild-type strain was transformed with FoSpc2 and FoSpc3 fluorescently labeled with GFP, and the strain transformed only with GFP was used as a negative control. Proteins were extracted as previously described (45). The protocol for AP-MS was as previously described (41).

### Generation of GFP fusion constructs.

To generate the FoSpc1-, FoSpc2-, FoSpc3-, and FoSec11-GFP constructs by gap repair, the entire signal peptidase gene, including its promoter region, was amplified using the primers listed in Table S1, cloned into a pKNT GFP vector using a one-step cloning kit (Vazyme Biotech, China), and verified by sequence analysis (46). AmyB-GFP and FoWc2-GFP were constructed using the same method.

### BiFC assays.

For BiFC assays, the full-length *FoSPC3*, *FoSPC2*, *FoSPC1*, and *FoSEC11* sequences were amplified and cloned into pCX62 vectors carrying CYFP and hygromycin resistance genes, and pKNT-NYFP vectors carrying NYFP and neomycin resistance genes. All constructs were verified by sequencing and cotransformed in pairs (as shown in the figure legends) into the wild-type protoplasts. YFP fluorescence signals in the transformants were observed using a Nikon A1R laser scanning confocal microscope (emission, 525/40 nm).

### Quantitative real-time PCR.

Total RNA was extracted using an Eastep Super total RNA extraction kit (Promega) from mycelia of the various strains harvested from liquid CM cultures after incubation at 28°C with constant shaking at 180 rpm for 2 days under different light conditions. cDNA was synthesized using a test kit and a reverse transcription kit (Vazyme). Quantitative PCR was carried out using a SYBR kit (TaKaRa) with specific primers (Table S1). The actin gene (Table S1) was used as the endogenous reference gene in this experiment. The data generated were calculated using the 2^−ΔΔ^*^CT^* method as previously reported (47). All experiments were independently repeated three times for each sample.
