# Supplementary material for: The Signal Peptidase FoSpc2 Is Required for Normal Growth, Conidiation, Virulence, Stress Response, and Regulation of Light Sensitivity in Fusarium odoratissimum
Source: Microbiol Spectr. 2023 Jun 27;11(4):e04403-22. doi: 10.1128/spectrum.04403-22 (PMC10433827; doi:10.1128/spectrum.04403-22)
Supplement: Supplemental file 1 — Supplemental material. Download spectrum.04403-22-s0001.docx, DOCX file, 0.2 MB [file spectrum.04403-22-s0001.docx]

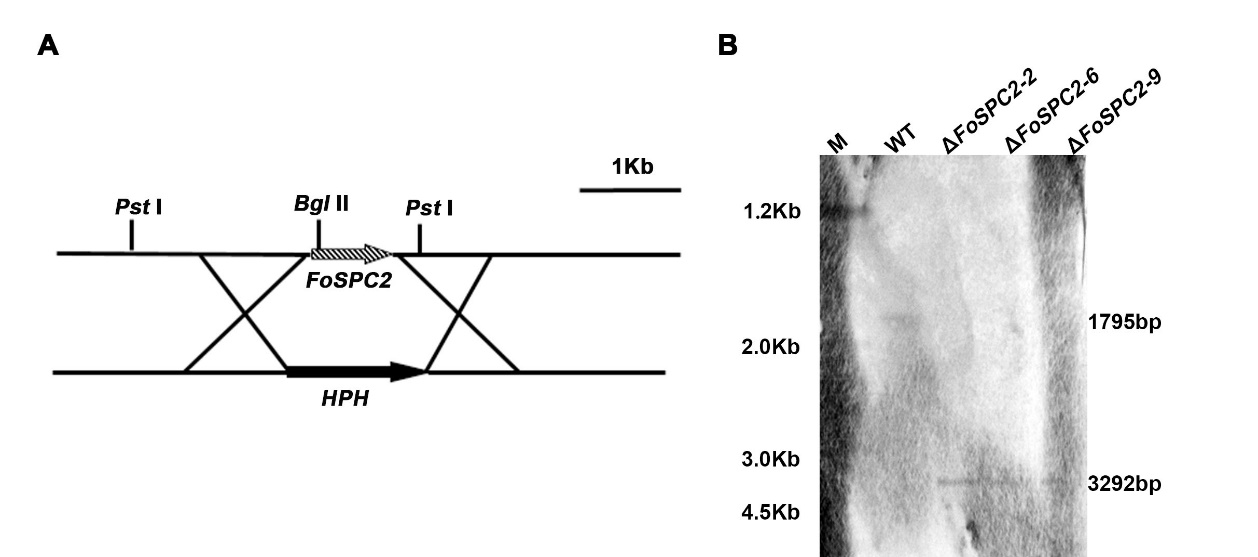


**FIG S1** The strategy of homologous recombination used to generate the deletion mutant and the Southern blot analysis of ∆*FoSPC2*. Genomic DNA of the strains was digested with *Pst* I and *Bgl* II. The upstream flanking sequences were used as probes.


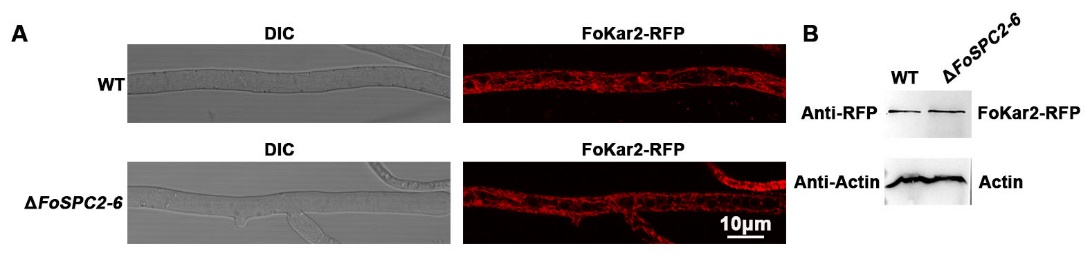


**FIG S2** Deletion of *FoSPC2* did not affect the maturation of FoKar2. (A) The subcellular localization of FoKar2 in wild-type (WT) and Δ*FoSPC2* strains. Bar=10μm. (B) The protein levels of FoKar2 in WT and Δ*FoSPC2* strains were detected by western blot.

**TABLE S1 Primers used in this study**

| Name | **Sequences (5′–3′)** |
| --- | --- |
| *FoSPC1*-Up-F | CCAGGGCCTATCGTATGAG |
| *FoSPC1*-Up-R | CATTCATTGTTGACCTCCACTAGCTCCAGGATGATGATCGGGTGGGAG |
| *FoSPC1*-Do-F | GCAAAGGAATAGAGTAGATGCCGACCGCTCGCTGTGTCATGACCTC |
| *FoSPC1*-Do-R | CCAAATTCACTGTGAGTC |
| *FoSPC1*-Id-F | CGATTTACCTGATCAACAG |
| *FoSPC1*-Id-R | CGTGCCTCCTAGACCTAG |
| *FoSPC1*-NEST-F | CACGGTTAGCCAGACGAG |
| *FoSPC1*-NEST-R | CGCAACTGCACCATCAAG |
| FoSpc1-GFP-F | AGGGAACAAAAGCTGGGTACCGGAAGGTGTATTACGGACTC |
| FoSpc1-GFP-R | GCCCTTGCTCACCATAAGCTTTTGATTTCCTTTGCTTGCAA |
| *FoSPC2*-Up-F | GGTCAAGGAATTGCCAGATC |
| *FoSPC2*-Up-R | CATTCATTGTTGACCTCCACTAGCTCCAGCCTGCAATGGCTTGTGTCG |
| *FoSPC2*-Down-F | GCAAAGGAATAGAGTAGATGCCGACCGGCACTGACAGGACATTGTC |
| *FoSPC2*-Down-R | CATCTACGTCAGGAAATGACG |
| *FoSPC2*-Nest-F | CGAAGATGCAGAGGAAGTAC |
| *FoSPC2*-Nest-R | TGGTCAGACGATATAGCGG |
| *FoSPC2*-Id-F | CGCGACACAGATCTCAAG |
| *FoSPC2*-Id-R | CTTAGAGACGGTGACACGC |
| FoSpc2-GFP-F | AGGGAACAAAAGCTGGGTACCCGCCTTGCGTCGCTTGCC |
| FoSpc2-GFP-R | GCCCTTGCTCACCATAAGCTTCGCCTTGCGTCGCTTGCC |
| *FoSPC3*-Up-F | GACTGACAATCCGTGATTTC |
| *FoSPC3*-Up-R | CATTCATTGTTGACCTCCACTAGCTCCATGATTAGCTGCAGTTGCAGC |
| *FoSPC3*-Down-F | GCAAAGGAATAGAGTAGATGCCGACCGGCATTCATAGTGTAATATGG |
| *FoSPC3*-Down-R | CGGGTAACTTACGTCTCTGC |
| *FoSPC3*-Nest-F | GTCAGCAGGAACATTTCAC |
| *FoSPC3*-Nest-R | GAACTGTTGCTGGTATCAGC |
| *FoSPC3*-Id-F | GCTGGCATATTCCAGAAGAC |
| *FoSPC3*-Id-R | GCTTCACATCATCTGTCTCG |
| FoSpc3-GFP-F | AGGGAACAAAAGCTGGGTACCCGTCAATCTCGATAGGCGAC |
| FoSpc3-GFP-R | GCCCTTGCTCACCATAAGCTTGTAGCTCTTCTTGGGAGCAT |
| *FoSEC11*-Up-F | GGTGCTGTGATGTTAACCAAG |
| *FoSEC11*-Up-R | CATTCATTGTTGACCTCCACTAGCTCCAGTATATCGGTTTCGTGGAG |
| *FoSEC11*-Down-F | GCAAAGGAATAGAGTAGATGCCGACCGGTGGTGTATGTGACGGGAAG |
| *FoSEC11*-Down-R | TTGAGGCGTTGGATCAGAGC |
| *FoSEC11-*Nest-F | GTGCACGAGAGGCGATGCTA |
| *FoSEC11*-Nest-R | CCATGGATGGTGATCTAAGG |
| *FoSEC11*-Id-F | GAACGTGTCTTCTTGAGCTC |
| *FoSEC11*-Id-R | CGTACGAGGTAGTCCTGG |
| FoSec11-GFP-F | AGGGAACAAAAGCTGGGTACCCGTGGATCTCGTAATTGAGG |
| FoSec11-GFP-R | GCCCTTGCTCACCATAAGCTTTTCTCGCTGTAGAACCACCA |
| FoSpc1-NYFP-F: | AGGGAACAAAAGCTGGGTACCGGAAGGTGTATTACGGACTC |
| FoSpc1-NYFP-R: | CGTGGCGATGGAGCGAAGCTTTTGATTTCCTTTGCTTGCAA |
| FoSpc1-CYFP-F: | AGGGAACAAAAGCTGGGTACC GGAAGGTGTATTACGGACTC |
| FoSpc1-CYFP-R: | CTTGCAGGCCGGGCGAAGCTT TTGATTTCCTTTGCTTGCAA |
| FoSpc2-NYFP-F: | AGGGAACAAAAGCTGGGTACCGGACCAACACAAGCCGAAG |
| FoSpc2-NYFP-R: | CGTGGCGATGGAGCGAAGCTTCGCCTTGCGTCGCTTGCCAC |
| FoSpc3-CYFP-F: | AGGGAACAAAAGCTGGGTACCCGTCAATCTCGATAGGCGAC |
| FoSpc3-CYFP-R: | CTTGCAGGCCGGGCGAAGCTTGTAGCTCTTCTTGGGAGCAT |
| FoSec11-CYFP-F: | AGGGAACAAAAGCTGGGTACCCGTGGATCTCGTAATTGAGG |
| FoSec11-CYFP-R: | CTTGCAGGCCGGGCGAAGCTTTTCTCGCTGTAGAACCACCA |
| FoSec11-NYFP-F: | AGGGAACAAAAGCTGGGTACCCGTGGATCTCGTAATTGAGG |
| FoSec11-NYFP-R: | CGTGGCGATGGAGCGAAGCTTTTCTCGCTGTAGAACCACCA |
| FoAmyB-F | AGGGAACAAAAGCTGGGTACCGACATGTATGCCTGACCTAG |
| FoAmyB-R | GCCCTTGCTCACCATAAGCTTGCTAGAGGCAACCAAGACCT |
| HYG-F | TGGAGCTAGTGGAGGTCAACAATGAATG |
| HYG-R | GTATTGACCGATTCCTTGCGGTCCGAA |
| HY-F | GATGTAGGAGGGCGTGGATATGTCCT |
| HY-R | CGGTCGGCATCTACTCTATTCCTTTGC |
| RT-*FoACTIN*-F: | CGTGAGAAGATGACCCAGATT |
| RT-*FoACTIN*-R: | CACCAGAGTCCAGAACGATAC |
| RT-*FoWC1*-F: | GCACAAGCAATCATGGGATAAG |
| RT-*FoWC1*-R: | CTTGCAAGACGGAGAGAGATATAG |
| RT-*FoWC2*-F | GGTCAGGACAACGATCTTATGG |
| RT-*FoWC2*-R | CGCTCATGTTGGAGTCATCT |
| RT-*FoVVD*-F: | CCCGTCACCAACTACAAGAAA |
| RT-*FoVVD*-R: | CTCAGAAAGCCGATGCAGTAA |
| RT-*FoPYH1*-F | CGCCAAGCACTCTCTAATGT |
| RT-*FoPYH1*-R | GCCTGTCGATCTTTGACTTCT |
| RT-*FoNOP1*-F | CGCTCTATGGGTTCTCTTTGT |
| RT-*FoNOP1*-R | GTGAGGGTTGTGAGGAAGTAAA |
| RT-*FoPHR*-F | CCACTAAGAGCCTTGCGATATT |
| RT-*FoPHR*-R | GTTCTCTTGGGAGGCATTGT |
| RT-*FoCRY*-F | GCGGGACTACATGCGATTAT |
| RT-*FoCRY*-R | GAGCGGTCTTCCACTTCTTATC |
| FoKar2-mCherry-F： | AGGGAACAAAAGCTGGGTACCGAACTCCGACCTGCAACAAG |
| FoKar2-mCherry-R： | CTCGGTGTTGTCCATAAGCTTAAGCTCATCATGGATGTTGTC |
|  |  |
